# A retrospective analysis of inotuzumab ozogamicin usage in adult patients with relapsed/refractory B-cell acute lymphoblastic leukemia

**DOI:** 10.3389/fonc.2025.1601782

**Published:** 2025-09-10

**Authors:** Narendra Agrawal, Pavan K. Boyella, Rahul Bhargava, Chandran K. Nair, Arijit Nag

**Affiliations:** ^1^ Department of Hemato-Oncology, Rajiv Gandhi Cancer Institute & Research Centre, New Delhi, India; ^2^ Department of Medical Oncology, Basavatarakam Indo American Cancer Hospital & Research Institute, Hyderabad, India; ^3^ Department of Hematology and Bone Marrow Transplant (BMT), Fortis Memorial Research Institute, Haryana, India; ^4^ Department of Clinical Hematology & Medical Oncology, Malabar Cancer Center, Kerala, India; ^5^ Department Clinical Hematology and Cellular Therapies, Tata Medical Center, Kolkata, India

**Keywords:** B-cell acute lymphoblastic leukemia, inotuzumab ozogamicin, CD22, antibody-drug conjugate, HSCT

## Abstract

**Introduction:**

The treatment of B-cell acute lymphoblastic leukemia (ALL) in India involves multi-agent chemotherapy and central nervous system (CNS) prophylaxis, but relapsed/refractory (R/R) B-cell ALL presents with high mortality and limited salvage options. Inotuzumab ozogamicin (InO), a monoclonal antibody targeting cluster of differentiation-22 (CD22) conjugated to calicheamicin, has improved outcomes for R/R B-cell ALL. A phase 3 multicenter trial (INO-VATE) in adult patients with CD22+ B-cell ALL has shown that InO significantly increases response rates and overall survival (OS) compared to standard treatments, with ongoing studies assessing its real-world effectiveness in India.

**Methodology:**

A multicentric, retrospective, observational study was conducted across five oncology centers in India to evaluate the effectiveness of InO in adult patients with R/R B-cell ALL. The study aimed to assess clinical outcomes, including the rate of complete remission (CR)/CR with incomplete hematologic recovery (CRi), minimal residual disease (MRD) negativity, duration of remission (DOR), OS, and the safety and tolerability of InO in real-world settings.

**Results:**

The medical records of adult patients (n = 32) aged >18 years with R/R B-cell ALL treated with InO between February 2017 and October 2022 were assessed. Among the total study participants, 59.4% (n = 19) achieved CR/CRi. Of these responders, MRD negativity was achieved in 94.7% (n = 18/19), and 68.4% (n = 13/19) achieved deep responses (MRD negative CR/CRi) after a median of two cycles of InO. The median DOR for those achieving CR/CRi was 6 months. Of the entire cohort, 34.4% (11/32) of the participants proceeded to hematopoietic stem cell transplantation (HSCT). The OS rates at 6 and 12 months in the entire cohort were 46.9% and 28.1%, respectively. The median relapse-free survival (RFS) among the responders was 7 months. Grade 3/4 treatment-related liver toxicity following InO initiation was reported in 37.5% of the participants. Myelosuppression-related adverse events were observed in 87.5% of the recipients.

**Conclusion:**

The real-world study (RWS) highlights the effectiveness of InO in achieving remission and MRD negativity in R/R B-cell ALL, although treatment-related toxicities remain a concern.

## 1 Introduction

Adult acute lymphoblastic leukemia (ALL) is the second mostxcommon leukemia in adults (1), with a prevalence of 20% of all cases of ALL (2). The treatment of adult ALL comprises multiple chemotherapy agents and allogeneic hematopoietic stem cell transplantation (HSCT) (3). Although the initial response rates with the conventional chemotherapeutic agents remain approximately 60% to 90%, only 30% to 50% maintain long-term disease-free survival (2).

After intensive multi-agent cytotoxic chemotherapy, the lack of minimal residual disease (MRD) in the marrow is the most significant and predictive indicator of a better clinical result in B-cell ALL patients (4). Cluster of differentiation-22 (CD22) is expressed in ≥90% of leukemic lymphoblasts and >90% of patients with B-cell ALL (3). Therefore, the use of an anti-CD22 antibody to treat B-cell malignancies was introduced (3).

Inotuzumab ozogamicin (InO) is an anti-CD22 antibody conjugated to calicheamicin and has been approved for use in relapsed/refractory (R/R) B-cell ALL based on the results of the INO-VATE trial (3, 4). The rate of complete remission (CR)/CR with incomplete hematologic recovery (CRi) was 73.8% [95% confidence interval (CI), 72%–88%] among patients who received InO monotherapy versus 29.4% (95% CI, 21%–39%) in the standard-of-care (SOC) chemotherapy arm (SC group; *p* < 0.001). Higher MRD negativity status, progression-free survival (PFS), and 2-year overall survival (OS) were also achieved in patients treated with InO (3). Additionally, a greater number of patients proceeded to HSCT with InO therapy.

In the Indian setting, the management of relapsed/refractory B-cell ALL is often complicated by delayed diagnosis, financial constraints, limited access to novel agents, and delays in drug procurement, particularly in public or resource-limited healthcare systems. These factors can lead to delayed initiation of salvage therapies, resulting in patients often presenting with higher disease burden and having received multiple prior lines of treatment by the time targeted therapies such as InO are introduced (2).

InO treatment for patients with R/R B-cell ALL was introduced in India in 2017 as a part of an early access program but was made commercially available in 2020. A single study of eight patients diagnosed with R/R B-cell ALL who had failed at least two prior lines of therapy (including the persistence of MRD), having received InO under a compassionate use program in India, concluded that InO-based salvage therapy improves the remission status in these patients (2). The real-world data on the use of InO in cases of adult R/R B-cell ALL in India are limited.

## 2 Objective

A multicentric, retrospective cohort analysis aimed to descriptively evaluate the clinical characteristics of adults diagnosed with CD22+ R/R B-cell ALL, treated with InO, and their associated clinical outcomes, in real-world practice in India.

## 3 Methods

This multicenter, retrospective study included adults (≥18 years) with R/R B-cell ALL treated with InO monotherapy from five oncology centers in India with confirmed CD22 positivity. Ph+ B-cell ALL patients were included only if they had failed ≥1 tyrosine kinase inhibitor (TKI) in addition to chemotherapy. The study cohort included patients who had been treated with the drug available through the early access program. The patient’s medical records were reviewed and collected by the participating oncologist and the research coordinators. The data were de-identified and sent to the study team for review and analysis. The data were collected until at least 6 months of follow-up were completed, extending to the latest available follow-up records. The primary endpoint was CR or CRi following treatment with InO on the basis of the number of salvage therapies (1 and ≥2) received before the initiation of InO. CR was defined as ≤5% blasts in the bone marrow, no evidence of extramedullary disease (EMD), and full recovery of peripheral blood counts (platelets > 100 × 10^9^/L and absolute neutrophil count > 1 × 10^9^/L). CRi was defined as patients who attained CR but had incomplete recovery of peripheral blood counts (platelets < 100 × 10^9^/L or absolute neutrophil count < 1 × 10^9^/L). The key secondary endpoints were MRD negativity rates, probability of OS and relapse-free survival (RFS) at 6 and 12 months, and analysis of grade 3/4 treatment-emergent adverse events (TEAEs) [including veno-occlusive disease (VOD)].

### 3.1 Statistical analyses

Mean, median, and standard deviations were provided for continuous variables while performing descriptive analysis of continuous data. Numbers and percentages were provided for dichotomous and polychotomous variables while performing descriptive analysis of categorical data. The proportion of CR/CRi achieved was observed across the number of salvage therapies before InO initiation, with respective 95% CI. Survival outcomes were analyzed using the Kaplan–Meier statistics. No statistical significance testing was performed due to the limited sample size (N = 32) and the retrospective, non-comparative design of the study. Given the heterogeneity in baseline characteristics and treatment history, formal hypothesis testing was considered inappropriate and unlikely to yield statistically meaningful conclusions. The analysis was therefore focused on descriptive evaluation to reflect real-world treatment patterns and outcomes, which may help guide future prospective studies with larger cohorts.

### 3.2 Ethics committee approval

Approval for this study was obtained from the respective institutional ethics committee (IEC)/institutional review board (IRB) before commencement of the study. The study was conducted in accordance with the approved study protocol and international ethical standards, including the Declaration of Helsinki and Indian regulatory requirements.

## 4 Results

**Figure 1:**
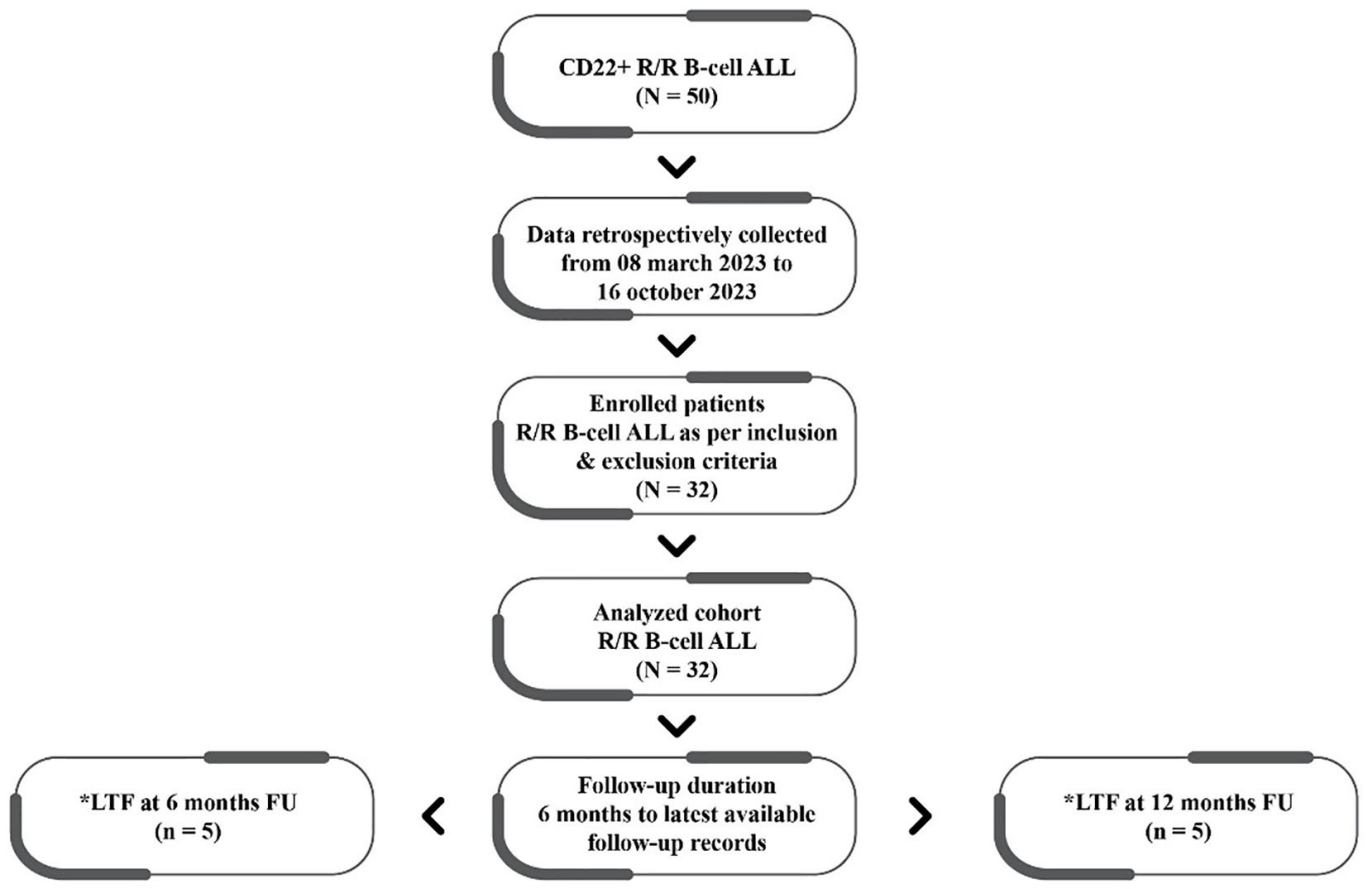
Patient disposition and follow-up. CD, cluster of differentiation-22; R/R, relapsed/refractory; ALL, acute lymphoblastic leukemia; LTF, lost to follow-up; FU, followed up.

The data of 32 patients (median age 37.41 ± 15.16 years) treated with InO were included in the final analyses. The patient disposition and follow-up are depicted in **Figure 1**, while the patient characteristics are detailed in **Table 1**.

**Table 1:** Patient characteristics.

Variable		Number of patients, N (%)
Participants enrolled	–	32
Gender	Female	11 (34.4)
	Male	21 (65.6)
Age	18–65 years	30
	>65 years	2
InO therapy received as salvage therapy	1	10 (31.3)
	2	18 (56.3)
	3	4 (12.5)
Number of total InO cycles	1	15 (46.9)
	2	8 (25)
	3	5 (15.6)
	4	2 (6.3)
	6	2 (6.3)
InO dosage for cycle 1	1.8 mg·m^−2^·cycle^−1^	12 (37.5)
	<1.8 mg·m^−2^·cycle^−1^	17 (53.1)
	>1.8 mg·m^−2^·cycle^−1^	3 (9.4)
Blast percentage in bone marrow at baseline	<50%	16 (50)
	>50%	16 (50)

InO, inotuzumab ozogamicin.

### 4.1 Patient characteristics

Prior to the initiation of InO, 28 patients had relapsed, while four patients were refractory to previous chemotherapy regimens. An equal number of patients (n = 16) had high- and low-burden diseases at the time of InO administration. A higher proportion of patients (56.4%; n = 18) had received InO as their second salvage and beyond. Approximately 37.5% (n = 12) had received InO in cycle 1 as per the recommended fractionated dose schedule of 0.8 mg/m^2^ on day 1, 0.5 mg/m^2^ on day 8, and 15mg/m^2^ in a 21–28-day cycle (dose = 1.8 mg·m^−2^·cycle^−1^).

### 4.2 Primary endpoints

#### 4.2.1 Response to InO therapy

Among the total study participants (n = 32), CR or CRi was achieved in 59.4% (19/32; CR = 18, CRi = 1). Most responses occurred after a median of two InO cycles (**Table 2**). The CR/CRi rate was higher in patients receiving InO as the first (60%) or second (61%) salvage therapy compared to later lines (50%) (**Table 3**). Remission was not achieved in 37.5% (n = 12/32), and one patient was lost to follow-up.

**Table 2:** Median number of cycles of InO needed to attain CR/CRi.

Number of patients	Median	Range
19	2	1 to 6

InO, inotuzumab ozogamicin; CR/CRi, complete remission with incomplete hematologic recovery.

**Table 3:** Number of patients achieving CR/CRi as per the salvage therapy with InO.

Salvage	Number of patients (N = 32)	CR/CRi (n [%])
First salvage	10	6 (60)
Second salvage	18	11 (61)
Third salvage	4	2 (50)

InO, inotuzumab ozogamicin; CR/CRi, complete remission with incomplete hematologic recovery.

CR/CRi rates were comparable among the patients with <50% (62.5%) and >50% (56.25%) blasts in baseline bone marrow studies [bone marrow blast (BMB)] (**Table 4**), indicating that the rate of response was not influenced by the disease burden.

**Table 4:** Number of patients achieving CR/CRi following treatment with InO classified as per high-burden and low-burden disease.

Disease burden at baseline	Number of patients (N [%]) N = 32	CR/CRi (N [%])
^$^BMB < 50%	16 (50)	10 (62.5)
^$^BMB > 50%	16 (50)	9 (56.25)

^$^BMB, bone marrow blast; InO, inotuzumab ozogamicin; CR/CRi, complete remission with incomplete hematologic recovery.

### 4.3 Secondary endpoints

#### 4.3.1 MRD negativity

Among 19 patients (59.4%) who achieved CR or CRi, 94.7% (18/19) attained MRD negativity following InO therapy (**Table 5**).

**Table 5:** Number of patients achieving MRD negativity following initiation of InO among those who had CR/CRi.

Number of patients (N = 19)	Achieved CR N (%)	Achieved CRi N (%)	Patients attaining CR + CRi
	18	1	19
Attained MRD negativity	17 (94.4)	1 (100%)	18 (94.8)
Not attained MRD negativity	1 (5.6)	0	1 (5.2)

MRD, minimal residual disease; InO, inotuzumab ozogamicin; CR/CRi, complete remission with incomplete hematologic recovery.

#### 4.3.2 HSCT and rate of transplantation

Among the responders (CR/CRi), 47.4% (9/19) proceeded to HSCT, and 66.7% (6/9) underwent HSCT after a single cycle of InO.

#### 4.3.3 The median DOR

Based on available data from 17 study participants, the median duration of remission (DOR) was 6 months in the patients who attained CR/CRi.

#### 4.3.4 Overall survival

**Figure 2:**
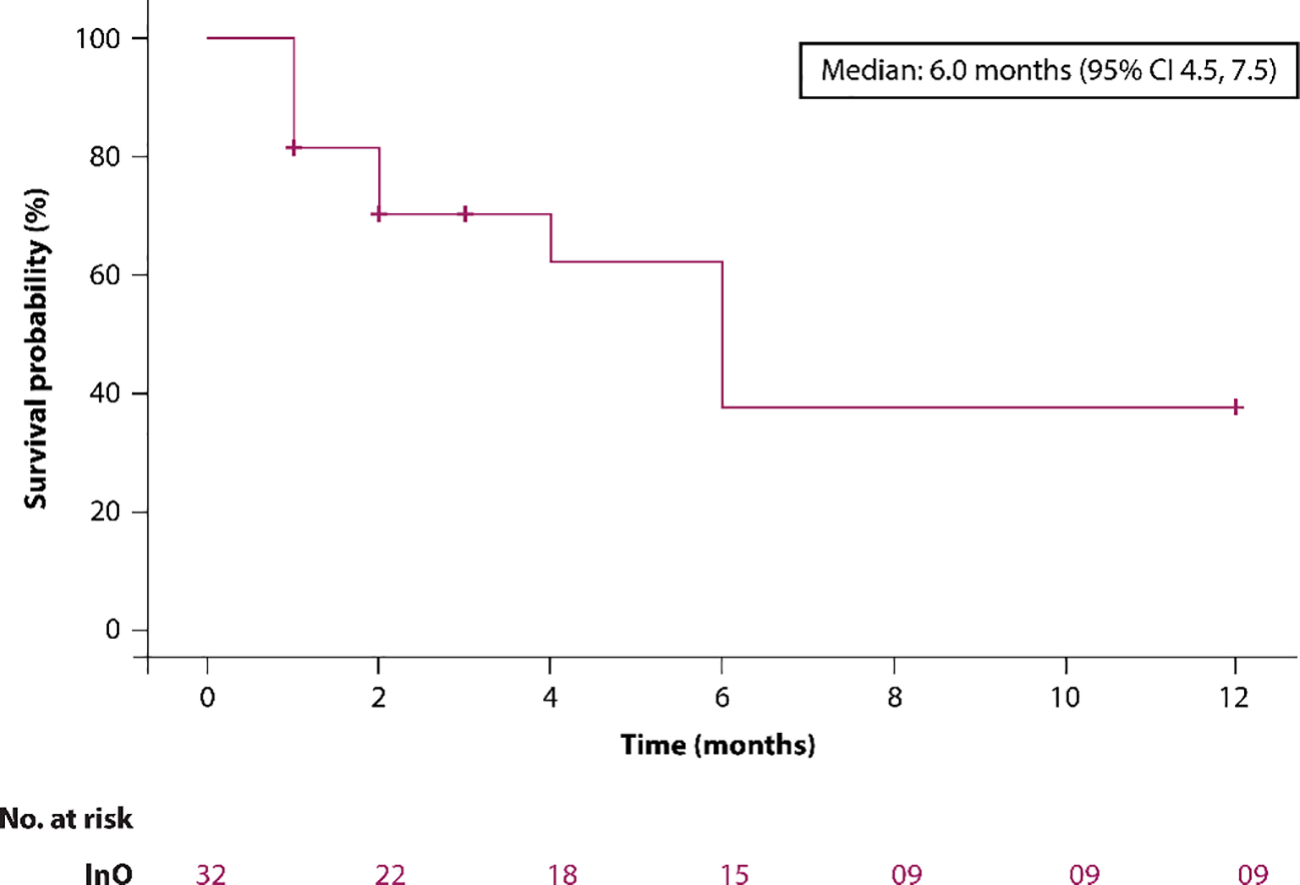
The Kaplan–Meier survival curve.

Overall survival (OS) at 6 and 12 months was 46.9% and 28.1%, respectively. The median OS was 6 months (95% CI: 4.5–7.5) depicted in **Figure 2**. In patients aged >65 years (n = 2), the 6- and 12-month OS rates were both 50%.

#### 4.3.5 RFS

The median RFS in patients who achieved CR/CRi was 7.0 months (95% CI: 6.1–8.9 months).

#### 4.3.6 EMD

Of the 32 patients in the cohort, 6.25% (n = 2/32) had EMD at baseline. One of them achieved CR/CRi with InO and had subsequently undergone HSCT. Another patient did not achieve remission.

#### 4.3.7 Safety of InO

In the overall cohort, VOD was observed in 22% of patients (n = 7/32) receiving InO, and 71.4% of these patients (n = 5) developed VOD after HSCT (**Table 6**). Grade 3/4 hepatotoxicity occurred in 37.5% of patients, while hematologic toxicities were the most frequent adverse events, affecting 87.5% of the cohort (**Table 7**).

**Table 6:** Proportion of patients with adverse events having undergone HSCT and not undergone HSCT.

Patient status	Number of patients having undergone HSCT, n (%)	Number of patients not having undergone HSCT, n (%)
VOD	5 (45.5)	2 (10)
Grade 3/4 liver toxicities	4 (36.4)	8 (40)
Hematological toxicities	9 (81.8)	19 (95)

HSCT, Hematopoietic stem cell transplantation; VOD, veno-occlusive disease.

**Table 7:** Proportion of patients with VOD by number of salvage therapies.

Patient status	Number of patients	N (%)
InO as first salvage therapy	10	2 (20)
InO as second salvage therapy	18	3 (16.8)
InO as third salvage therapy	4	2 (50)

InO, inotuzumab ozogamicin; VOD, veno-occlusive disease.

#### 4.3.8 Cause of death

There were 17 deaths in the cohort (53.1%). Disease progression (60.8%) was the major cause of mortality. Septic shock was responsible for 11.8% of deaths. VOD-related complications, including multi-organ failure and VOD-associated pneumonia, each accounted for 5.9% of deaths, both occurring in patients with a history of HSCT. Central nervous system (CNS) relapse contributed to 5.9% of fatalities, while the cause of death was not documented in 11.8% of cases (**Table 8**).

**Table 8:** Adverse events and cause of death.

Hematological toxicities (87.5% [n = 28/32])	
Thrombocytopenia	27 (84.8)
Neutropenia	20 (62.5)
Febrile neutropenia	14 (43.7)
Grade 3/4 treatment-related liver toxicities (37.5% [n = 12/32])	
Increased aspartate aminotransferase	9 (28.1)
Increased alanine aminotransferase	6 (18.8)
Increased bilirubin	8 (25)
Cause of death (53.1% [n = 17/32])	
Progression of disease	4 (23.5)
Relapse	6 (35.3)
Septic shock	2 (11.8)
VOD-related multi-organ failure	1 (5.9)
VOD pneumonia	1 (5.9)
CNS relapse	1 (5.9)
Not mentioned	2 (11.8)

VOD, veno-occlusive disease; CNS, central nervous system.

## 5 Discussion

InO was effective in adults with R/R B-cell ALL, with 59.4% of the patients achieving CR/CRi, and most of these patients achieved MRD negativity (94.7%). Overall, CR/CRi was achieved after a median of two cycles of InO. The global, phase 3 INO-VATE ALL study also concluded that InO achieved higher response rate (CR/CRi [73.8% {95% CI, 72%–88%} versus 29.4% {21%–39%}; *p* < 0.001]) and MRD-negativity rates (78.4% [68%–87%] versus 28.1% [14%–47%]; *p* < 0.001) compared to standard chemotherapeutic regimens in patients with R/R B-cell ALL (3). Interestingly, the CR/CRi rate reduced to 66.7% among those receiving InO as salvage 2 in INO-VATE ALL (3). **Table 9** summarizes the results of various InO trials.

**Table 9:** Clinical trials with InO.

Study	Phase	Cohort (n)	Dosing	CD22 positivity	CR/CRi	Median OS (months)	Duration of remission (months)
Kantarjian et al., 2012 (12)	II	Adults and children with R/R ALL (49)	1.3–1.8 mg/m^2^ q3–4 weeks	>50%	CR 9/49 (18%) CRi 4/49 (8%)	5.1 (responders)	—
Kantarjian et al., 2013 (11)	II	R/R ALL (90)	1.3–1.8 mg/m^2^ q3–4 weeks or 0.8 mg/m^2^ (day 1), then 0.5 mg/m^2^ (days 8 and 15) q3–4 weeks	>50%	CR 17/90 (19%) CRi 8/90 (19%	6.2	7
Advani et al., 2014 (8)	II	R/R ALL (35)	0.8 mg/m^2^ (day 1), then 0.5 mg/m^2^ (days 8 and 15	99%	CR 11/35 (31%) CRi 12/35 (34%)	7.4	—
Kantarjian et al., 2016 (13)	III	R/R ALL (326)	0.8 mg/m^2^ (day 1), then 0.5 mg/m^2^ (days 8 and 15) q3–4 weeks	74/109 (68%) >90% CD22 positive	CR 39/109 (36%) CRi 19/109 (17%)	7.7	4.6
Torrent et al., 2023 (7)	RWS	R/R ALL (34)			CR/CRi 64%	4	3.5
Present retrospective	RWS	R/R ALL (32)	0.8 mg/m^2^ (day 1), then 0.5 mg/m^2^ (days 8 and 15) q3–4 weeks	100%	CR/CRi 59.4%	—	6

InO, inotuzumab ozogamicin; RWS, real-world study; CR/CRi, complete remission with incomplete hematologic recovery; R/R, relapsed/refractory; ALL, acute lymphoblastic leukemia; CD, cluster of differentiation-22; OS, overall survival.

In the present retrospective real-world study (RWS), the remission rate may have been slightly lower compared to that of the phase 3 study due to multiple reasons. The sample size was smaller compared to that of INO-VATE ALL. Therapy with InO was started later in the course of the disease due to logistic constraints, resulting in a more heavily pre-treated cohort of patients. Previous studies have reported a lower response rate with InO when it is started late in 2+ treatment lines. Similarly, in the present RWS, a greater number of patients achieved CR/CRi with InO as first- and second-line salvage treatments as compared to later lines (5–7).

A retrospective study with InO concluded remission in all seven evaluable patients, and six (85.7%) of them also achieved MRD-negative status (2). A phase II study evaluated the safety and efficacy of InO as a standard dose of 1.8 mg·m^−2^·cycle^−1^ (0.8 mg/m^2^ on day 1 and 0.5 mg/m^2^ on days 8 and 15) in patients with CD22+ R/R B-cell ALL in the second and later salvage settings. The remission rate (CR/CRi) was 65.7%, while 78% (n = 18/23) of patients with CR/CRi achieved MRD negativity (8). In other studies, the CR/CRi was 63%, 53%, and 64% in patients diagnosed with R/R B-cell ALL and treated with InO in the second or later salvage setting (7, 9, 10). The remission rates of the present retrospective RWS are comparable to those of these studies.

The OS rate was 46.9% with a median DOR of 6 months for those achieving CR/CRi in the present retrospective RWS. In the INO-VATE study, the DOR was 5.4 months (11). The RFS period was 7 months in the present retrospective RWS, while PFS was 5.4 months in the INO-VATE study among those achieving CR/CRi (11). A retrospective RWS reported a median DOR of 11.5 months [95% CI, 11.5 to not reached (NR)]. The median OS after InO initiation was 11.6 months (95% CI, 7–15.4). When censored at the time of allo-HSCT, it was 13.6 months (95% CI, 5.5 to NR) (9).

The safety profile of InO was consistent with that of previous studies (2, 11). The VOD was a common non-hematologic toxicity. Higher rates of VOD were seen in the overall cohort compared to the INO-VATE study (21.87% vs. 11%). Higher rates of VOD were observed after HSCT. The use of myeloablative conditioning regimens coupled with a higher percentage of haploidentical HSCTs using the post-transplant cyclophosphamide backbone could have accounted for this. The incidence of non-VOD hepatic derangement was also higher in the current RWS. There is a high prevalence of steatohepatitis in India, which could have predisposed these patients to higher rates of hepatotoxicity with InO.

## 6 Limitations

This retrospective, multicentric study has inherent limitations of a RWS, including inconsistency in reporting and follow-up, resulting in missing data, particularly on MRD timing and toxicity. The non-randomized design involves potential selection bias, and the limited sample size restricts subgroup analysis and definite findings on comparative safety or efficacy. Despite these limitations, the study provides valuable real-world evidence on InO use in Indian clinical practice and emphasizes the importance of bigger, prospective trials.

## 7 Conclusion

In this real-world analysis, InO monotherapy was an effective and relatively safe agent for the management of adults with CD22+ R/R B-cell ALL. Larger prospective studies are needed to better characterize long-term outcomes of the patients treated with InO-based therapy.

## Data Availability

The original contributions presented in the study are included in the article/supplementary material. Further inquiries can be directed to the corresponding author.
